# Parafoveal Previews and Lexical Frequency in Natural Reading: Evidence From Eye Movements and Fixation-Related Potentials

**DOI:** 10.1037/xge0000494

**Published:** 2018-10-18

**Authors:** Federica Degno, Otto Loberg, Chuanli Zang, Manman Zhang, Nick Donnelly, Simon P. Liversedge

**Affiliations:** 1 Centre for Vision and Cognition, School of Psychology, University of Southampton; 2 Department of Psychology, University of Jyväskylä; 3 Academy of Psychology and Behavior, Tianjin Normal University; 4 School of Psychology, University of Southampton; 5 School of Psychology, University of Central Lancashire

**Keywords:** eye movements, fixation-related potentials, parafoveal-on-foveal effects, preview effects, lexical frequency

## Abstract

Participants’ eye movements and electroencephalogram (EEG) signal were recorded as they read sentences displayed according to the gaze-contingent boundary paradigm. Two target words in each sentence were manipulated for lexical frequency (high vs. low frequency) and parafoveal preview of each target word (identical vs. string of random letters vs. string of Xs). Eye movement data revealed visual parafoveal-on-foveal (PoF) effects, as well as foveal visual and orthographic preview effects and word frequency effects. Fixation-related potentials (FRPs) showed visual and orthographic PoF effects as well as foveal visual and orthographic preview effects. Our results replicated the early *preview positivity* effect (Dimigen, Kliegl, & Sommer, 2012) in the X-string preview condition, and revealed different neural correlates associated with a preview comprised of a string of random letters relative to a string of Xs. The former effects seem likely to reflect difficulty associated with the integration of parafoveal and foveal information, as well as feature overlap, while the latter reflect inhibition, and potentially disruption, to processing underlying reading. Interestingly, and consistent with Kretzschmar, Schlesewsky, and Staub (2015), no frequency effect was reflected in the FRP measures. The findings provide insight into the neural correlates of parafoveal processing and written word recognition in reading and demonstrate the value of utilizing ecologically valid paradigms to study well established phenomena that occur as text is read naturally.

The investigation of the time course of visual word recognition has been of special interest for researchers in the field of cognitive science. Indeed, it is of particular importance to understand how the brain represents and transforms information during reading, and the nature of processes that occur as different stages of processing unfold over time, from the early visual perception of a word form through to its full identification and linguistic interpretation.

Although there has been a large amount of research conducted with both eye movements and event-related potentials (ERPs) to examine the timing of the processes underlying reading, the debate on the exact time course of these processes is still ongoing. As suggested by Laszlo and Federmeier (2014), the time course of visual word recognition may vary under different circumstances, and this may be one of the reasons for the inconsistency of results. Indeed, ERP experiments have traditionally investigated reading by presenting words one at a time, while eye movement research investigating written text comprehension has focused on reading of words in context.

It is evident that the paradigm typically used in ERP research (i.e., Rapid Serial Visual Presentation [RSVP]), in which one word at the time is presented in the center of the screen, is different from natural reading conditions in several ways. First, participants are required to fixate the middle of the screen and avoid making any eye movements (which is itself quite unnatural in relation to normal reading). Second, the required central fixation and successive word-by-word presentation prevents some natural eye movement phenomena from occurring; for example, word skipping, natural refixations, and regressions. Third, each word is displayed on the screen for a set amount of time (typically between 400 ms and 1,000 ms), preventing readers having control over how long they fixate each word. Fourth, the speed of reading is reduced, as the rate with which words are presented on the screen is typically slower than the natural reading pace. Lastly, parafoveal information is not available. This last point is of crucial importance. A distinctive feature of natural reading is that multiple words are presented in one, or more, horizontal lines of text and the words are processed (largely) successively, in the order in which they appear. This means that during natural reading both foveal (i.e., the central 2° of the visual field) and parafoveal (i.e., 2°–5° of the visual field) information is available during any particular fixation. The important point to note here is that visual information about a word is encoded during two separate perceptual phases; first, visual degraded information about a word is accrued over time when the word is in the parafovea (parafoveal processing); second, nondegraded visual information about the constituent letters of a word becomes rapidly available when the word is directly fixated (foveal processing). To be clear, then, the RSVP technique, by its very nature, cannot deliver visual information in a manner that accurately approximates the way visual information is delivered during natural reading. Thus, the ERP studies conducted to date using RSVP do not permit researchers to investigate parafoveal processing, nor to examine the time course of foveal processing of a word in relation to preceding parafoveal processing of that word. In short, RSVP does not permit the study of perceptual and linguistic processing as it occurs naturally during reading.

## Parafoveal Processing

Aiming to overcome some of the limitations associated with ERP investigations that adopt an RSVP methodology, two approaches have been developed: (a) the use of RSVP-with-flanker-word-presentation, and (b) the coregistration of eye movements and EEG signal. In the first approach, sentences or lists of words are presented one by one in the center of the screen, and are simultaneously flanked to the left and to the right of the central fixation with the preceding and following word(s) in the text (Barber, Ben-Zvi, Bentin, & Kutas, 2011; Barber, Doñamayor, Kutas, & Münte, 2010; Barber, van der Meij, & Kutas, 2013; Kornrumpf, Niefind, Sommer, & Dimigen, 2016; Li, Niefind, Wang, Sommer, & Dimigen, 2015; Niefind & Dimigen, 2016). It is argued that this method provides a more natural reading situation than the traditional RSVP approach, as parafoveal information is available; however, the fact remains that this approach is artificial because it still requires that readers maintain central fixation. Thus, readers are unable to engage in skipping, natural refixations, and regressive oculomotor behavior. Furthermore, because it is well documented that attention is allocated to the location of saccade targets prior to the initiation of those saccades, it is very likely the case that this paradigm does not cause readers to process parafoveal information attentionally in the manner they do during normal reading. In our view, this approach does not effectively circumvent all the shortcomings of the more standard RSVP paradigm.

In the second approach, readers are free to move their eyes, and eye movements and EEG signal are simultaneously recorded while participants read pairs of words (Baccino & Manunta, 2005; López-Peréz, Dampuré, Hernández-Cabrera, & Barber, 2016; Simola, Holmqvist, & Lindgren, 2009), lists of words (*saccadic reading*; Dimigen et al., 2012; Hutzler et al., 2007, 2013; Kornrumpf et al., 2016; Niefind & Dimigen, 2016), sentences (Dimigen, Sommer, Hohlfeld, Jacobs, & Kliegl, 2011; Kretzschmar, Bornkessel-Schlesewsky, & Schlesewsky, 2009; Kretzschmar et al., 2015; Metzner, von der Malsburg, Vasishth, & Rösler, 2016; Weiss, Knakker, & Vidnyánszky, 2016), or paragraphs (Henderson, Luke, Schmidt, & Richards, 2013). This technique allows for registration of continuous brain activity over time under more standard reading conditions, as participants make natural eye movements as they process the text. In addition, this method allows the experimenter to time-lock the ERPs to particular oculomotor events—for example, to a particular fixation onset on a critical word in the sentence. These time-locked signals are known as fixation-related potentials (FRPs). This approach is also very valuable in that it offers sufficient flexibility that other, very useful, experimental methods, such as gaze-contingent paradigms can be used simultaneously to study reading (see Rayner, 1998, 2009 for reviews on this type of paradigms). One such gaze-contingent paradigm that has been widely used is the boundary paradigm (Rayner, 1975). In this paradigm an invisible boundary is embedded in the text. Prior to crossing the boundary, the target word is replaced by a preview stimulus. When the readers’ eyes cross the boundary, the preview stimulus is replaced by the target word. By manipulating the relationship between the preview stimulus and the target word, it is possible to study the type of information that readers extract from the parafovea. Measuring FRPs in boundary paradigm experiments provides an opportunity to investigate the neural correlates of parafoveal-on-foveal (PoF) effects and preview effects—two phenomena that are very central to current understanding of parafoveal processing.

### Parafoveal-on-Foveal Effects

PoF effects refer to any influence that the characteristics of parafoveal words have on processing of the currently fixated foveal word. Evidence of the existence of these effects is controversial both in the eye movement and FRP literature (Brothers, Hoversten, & Traxler, 2017; Drieghe, 2011; Hyönä, 2011). Moreover, examining PoF effects is critical to understand whether words are lexically processed in a serial or parallel fashion during reading. Serial processing accounts, for instance the E-Z reader model (Reichle, Pollatsek, Fisher, & Rayner, 1998; Reichle, Warren, & McConnell, 2009), suggest that attention is allocated serially such that words are sequentially fully identified one at a time. Thus, according to serial models, parafoveal processing of the upcoming word can be initiated before a saccade is made to that word, but only when lexical processing of the currently fixated word has been completed. This implies that, if word identification proceeds serially, preattentive parafoveal processing is limited to the extraction of sublexical features of the upcoming word (e.g., visual and orthographic properties). In contrast, parallel processing accounts, such as the SWIFT model (Engbert, Nuthmann, Richter, & Kliegl, 2005; Schad & Engbert, 2012), assume that attention is distributed over a spatially extended portion of text during reading such that all words within the perceptual span are simultaneously lexically processed. This means that parallel models predict that both sublexical and lexical PoF effects must occur (at least on a significant proportion of fixations). Previous research testing whether visual and orthographic properties, as well as the lexical frequency of parafoveal words, influence processing of the fixated word is reviewed below.

#### Visual and orthographic PoF effects

Some eye movement studies have reported PoF effects produced by unusual letter combinations in the parafovea (see Schotter, Angele, & Rayner, 2012 for a summary). Visually unusual and orthographically unfamiliar parafoveal previews lead to longer fixation times on the currently fixated word (e.g., Drieghe, Rayner, & Pollatsek, 2008; Inhoff, Starr, & Shindler, 2000; Rayner, 1975, cf. Pynte, Kennedy, & Ducrot, 2004; White, 2008). However, other studies have shown no such effects (e.g., Rayner, Juhasz, & Brown, 2007; White & Liversedge, 2004, 2006).

Similarly, the results are mixed in the FRP literature. Three studies using coregistration of eye movements and EEG signal have investigated the neural correlates of orthographic PoF effects. Baccino and Manunta (2005) presented pairs of prime-target words in a semantic relatedness decision task. They found that nonwords made up of illegal letter combinations elicited less negative amplitudes around 119 ms at left occipital sites and less positive amplitudes at around 140 ms at the right central and frontal electrodes compared with semantically related and unrelated target words, suggesting some foveal processing sensitivity to orthographic properties of parafoveal words (though the task was not natural reading). In a similar experiment, Simola, Holmqvist, and Lindgren (2009) presented prime-target pairs with the target words being displayed either in the right or left visual field. The authors found that illegal letter combinations presented in the right visual field elicited less positive amplitudes between 200 ms and 280 ms at occipital sites compared with semantically related and unrelated target words. Again, this experiment demonstrates a sensitivity to parafoveal orthographic information and that this exerts an influence on FRPs at fixation. In a third study, Dimigen, Kliegl, and Sommer (2012) presented list of words in a semantic category decision task. For any particular word in the list, the parafoveal word could be identical, semantically related or unrelated to the fixated word. In this experiment, unlike the previous experiments, the authors did not observe any type of PoF effects, and thus, as noted above, the evidence in relation to visual and orthographic PoF effects is mixed. In relation to the current experiment, note that neural correlates of visual and orthographic PoF effects have never been investigated during natural reading of sentences.

#### Lexical PoF effects

Even more disputed are the lexical PoF effects of word frequency. These effects have been mainly found in corpus analysis studies (e.g., Kennedy & Pynte, 2005; Kliegl, Nuthmann, & Engbert, 2006; Pynte & Kennedy, 2006; Schad, Nuthmann, & Engbert, 2010). In such studies, reading times on the foveal word are generally shown to be longer when parafoveal words are low compared with high-frequency words. However, eye movement studies employing experimental manipulations of the parafoveal word frequency (as opposed to corpus approaches) have not shown PoF effects (e.g., Brothers et al., 2017; Calvo & Meseguer, 2002; Henderson & Ferreira, 1993; Rayner, Fischer, & Pollatsek, 1998; Schroyens, Vitu, Brysbaert, & d’Ydewalle, 1999; White, 2008). Advocates of the serial processing position attribute the inconsistency of results to inaccurate saccade targeting (see Drieghe, 2011; Hyönä, 2011 for reviews). Lexical PoF effects could result from mislocated fixations—that is when planned saccades undershoot the critical word and land on the previous word, but attention is still allocated to the originally intended location. In contrast, advocates of the parallel processing models explain the inconsistency as a difference in the time course of lexical compared with other low-level PoF effects (Risse & Kliegl, 2012, 2014).

Two coregistration experiments have investigated the PoF effects of word frequency, and each has obtained different results. Niefind and Dimigen (2016) presented a list of unrelated words in a boundary paradigm, semantic category decision task. The preview of the upcoming word could be identical to the target word or a different word with the opposite word frequency to the target word (low vs. high or high vs. low). Early PoF effects of frequency were observed between 130 ms and 140 ms at two right-frontal electrodes (AF8 and F4), with more positive amplitudes for low-frequency than high-frequency parafoveal previews. Late PoF effects were detected between 630 ms and 640 ms at one left parietal electrode (CP5), with low-frequency parafoveal previews eliciting more negative amplitudes than high-frequency parafoveal preview stimuli. Kretzschmar et al. (2015) examined PoF effects of word frequency in a sentence reading task, with high- or low-frequency target words embedded in either a high or low predictability sentence context. The authors investigated the time window between 150 ms and 400 ms after fixation onset of the foveal word over centro-parietal sites, but they did not observe any PoF effect due to the parafoveal word frequency. It is worth pointing out that high- and low-frequency target words were embedded in the sentences in such a way that the number and length of words preceding the target differed between conditions. It therefore remains important to investigate whether PoF effects might occur in a natural sentence reading task when stimuli that are controlled for content as well as predictability across conditions are used. Furthermore, it remains an open question as to whether PoF effects might be present at electrode sites and during temporal windows beyond those examined by Kretzschmar et al. (2015).

### Visual and Orthographic Preview Effects

One of the most well-established findings in the eye movement literature on reading is the preview effect demonstrated using the boundary paradigm. In such an experiment, when readers receive a valid preview of the upcoming parafoveal word, the identification of that word during the subsequent fixation is facilitated compared with when readers receive an invalid preview. Eye movement studies across different languages have consistently reported that parafoveal previews that have some form of visual or orthographic overlap facilitate the speed of processing of the target word (see Schotter et al., 2012 for a review). It has been argued that such effects arise due to the integration of information across fixations (Cutter, Drieghe, & Liversedge, 2015). This explains why, when readers have a preview stimulus that is visually similar to the target word, the time readers spend looking at the target is shorter than when the preview is visually dissimilar from the target word (e.g., Pollatsek, Lesch, Morris, & Rayner, 1992; Rayner, Balota, & Pollatsek, 1986; see Hyönä, Bertram, & Pollatsek, 2004 for a summary). Visually similar previews share low-level visual properties with the target word; thus, the visual features activate consistent abstract letter identities of both the preview stimulus and, to some extent, of the target word. Furthermore, preview and target commonality with respect to abstract orthographic representations also results in facilitation. For example, when the preview stimulus shares the same letter identities with the target, there is no difference in the time readers fixate the target word regardless of whether the preview was presented in the same or a different case (e.g., wOrD vs. WoRd; McConkie & Zola, 1979; Rayner, McConkie, & Zola, 1980). In this situation, abstract letter identities do not change across fixations.

Neural correlates of preview effects have been observed across different studies using flanker-word-presentation and coregistration of eye movements and EEG signal. An early effect of preview has been shown between 140 ms and 300 ms after fixation onset on the target word. This effect has been called *preview positivity* and consists of an attenuation of the N1 component amplitude for identical compared with invalid previews, which is maximal over occipito-temporal sites. In addition, a late effect of preview has also been observed over the N400 component. Between 300 ms and 500 ms after fixation onset, identical previews yielded less negative amplitudes than invalid previews over midparietal electrodes. These effects have first been observed by Dimigen et al. (2012) in a category semantic decision task comparing identical previews (e.g., *blade*-*blade*) to invalid previews (semantically related, e.g., *knife*-*blade*, or semantically unrelated, e.g., *sugar*-*blade*), while participants read a list of five unrelated German words displayed horizontally across a presentation screen. The findings have been replicated in Chinese with the RSVP-with-flanker-word presentation approach (Li et al., 2015), and in German with different types of preview mask (identical vs. partially visible in parafovea, Kornrumpf et al., 2016; identical vs. invalid, Niefind & Dimigen, 2016) and regardless of participant’s display change awareness (Dimigen et al., 2012). However, these effects have not been established in natural sentence reading, as yet.

## Foveal Lexical Frequency Effects

As discussed, it is a well-established finding in the eye movement literature that words that occur with low frequency receive more and longer fixations compared with those that occur with high frequency (e.g., Inhoff & Rayner, 1986; Rayner & Duffy, 1986; see Rayner, 1998, 2009 for reviews). Note also that robust frequency effects have been reported to occur at the first fixation on a word (approximately 250 ms; see, e.g., Rayner & Duffy, 1986). However, while such effects may be apparent in eye movement measures by the end of the initial fixation on a word, the exact time course of the effects in ERPs is still a matter of debate. With respect to RSVP methodology, the most robust effects of frequency have been observed between 300 ms and 500 ms at central parietal and occipital sites (e.g., Dambacher & Kliegl, 2007; Hauk et al., 2006; Hauk & Pulvermüller, 2004; Münte et al., 2001; Osterhout, Bersick, & McKinnon, 1997; Rugg, 1990; Van Petten & Kutas, 1990; Young & Rugg, 1992), with N400 amplitudes decreasing with increased word frequency. Earlier effects have also been reported between 100 ms and 150 ms at central, parietal, and occipital electrodes (e.g., Dambacher et al., 2012; Hauk et al., 2006; Sereno, Rayner, & Posner, 1998), between 150 ms and 250 ms at fronto-central and occipital electrodes (e.g., Dambacher et al., 2012; Dambacher, Kliegl, Hofmann, & Jacobs, 2006; Hauk et al., 2006; Hauk & Pulvermüller, 2004) and between 280 ms and 335 ms at left anterior sites (e.g., King & Kutas, 1998). In addition, effects of target frequency have been observed at occipito-temporal and fronto-central electrodes between 200 ms and 300 ms in a RSVP-with-flanker-word-presentation experiment, and between 140 ms and 300 ms in a saccadic reading experiment (Niefind & Dimigen, 2016). Nonetheless, in the only study that investigated the neural correlates of lexical frequency during natural reading (Kretzschmar et al., 2015), in which centro-parietal electrode sites were examined between 150 ms and 650 ms after fixation onset, no significant effects were observed. One possibility for the null effects may be due to the electrode sites considered, as they were limited to centro-parietal areas, and results for occipito-temporal sites were not reported. Thus, it remains important to establish whether, by considering broader areas of the scalp, these effects may show their influence in natural reading, and if that is the case, whether they have an early or late onset.

## Current Research

**Figure 1 fig1:**
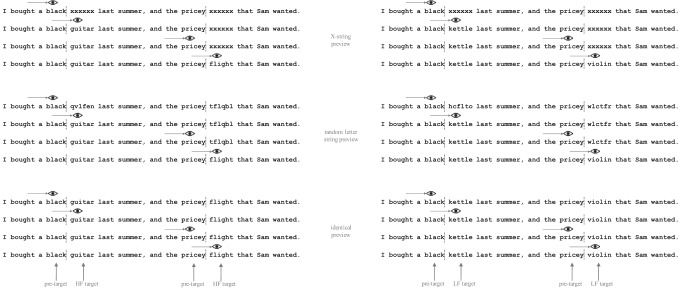
Illustration of the paradigm used. Participants read one-line sentences, and preview was manipulated for two words embedded in each sentence according to the gaze contingent boundary paradigm. When the participants’ eyes were looking at the pretarget word, the preview stimulus could be a string of Xs, a string of letters, or a word identical to the target word. Left panel: conditions with high-frequency target words (i.e., *guitar* and *flight*). Right panel: conditions with low-frequency target words (i.e., *kettle* and *violin*).

In the present study, we used coregistration of eye movements and EEG signal to investigate the time course of language processing under natural reading conditions, in which both foveal and parafoveal processing occur. Using the boundary paradigm, two target words in each sentence were manipulated for lexical frequency (high vs. low frequency)^1^ and parafoveal preview (X string preview vs. letter string preview vs. identical preview; see Figure 1). Participants read the single sentences for comprehension under normal reading conditions.[Fig-anchor fig1]

The objectives of the current experiment were a) to investigate the neural correlates of visual, orthographic, and lexical PoF effects; (b) to identify the neural correlates of the preview effect in natural reading, and if successful, to further demonstrate that those neural correlates may be modulated by the degree to which the preview is related to the target word; and (c) to explore possible neural correlates of the foveal lexical frequency effect under natural reading conditions (i.e., beyond the experimental situation considered by Kretzschmar et al., 2015).

With respect to the first objective, because an X-string preview shares very few features or letters with the target (or indeed with any word), then it is very visually dissimilar to a word, and it is likely that participants would detect the string in the parafovea (Angele, Slattery, & Rayner, 2016; Slattery, Angele, & Rayner, 2011). Thus, given its visual oddity and its likely parafoveal detection, disruption to processing may occur at the pretarget word (i.e., a visual PoF effect). An invalid preview formed of random letters (similar in shape to the letters of the target word) would have a less visually odd appearance in the parafovea, and therefore, processing at the pretarget word would be disrupted to a lesser degree (with any disruption reflecting an orthographic PoF effect). The identity preview condition, in contrast to the other two forms of preview, should produce no disruption to processing at the pretarget word. Finally, as discussed above, evidence for lexical PoF effects, as demonstrated by an effect of the lexical frequency of the preview at the pretarget word, is mixed in the literature. Certainly, a modulation of eye movements and FRPs based on the frequency of the parafoveal stimulus would provide support for a model of reading wherein more than the fixated word is fully identified in parallel. Alternatively, a lack of lexical PoF effects in the eye movement or in the FRP data would provide no evidence to support parallel processing, and would be consistent with a serial processing position.

As is standard in boundary paradigm experiments such as this, for the effects at the target word, we predicted increased preview benefit in the identity condition, less preview benefit in the random letter string preview condition, and least preview benefit in the X-string preview condition. This is the pattern of effects that we would certainly predict for the eye movement measures alone (based on a wealth of existing literature). Note, though, as mentioned earlier, a letter string preview is more word-like than a X-string preview, and therefore it is possible that the FRP data at the target may reflect increased difficulty associated with the integration of the target with letter string previews than X-string previews, as well reduced difficulty caused by feature overlap between target and preview. Importantly, this is the first study to use coregistration methodology to explore differences in FRPs between previews comprised of strings of letters and strings of Xs, and therefore, this is the first opportunity to explore whether different preview types have a differential influence on the time course of foveal processing (e.g., Hutzler et al., 2013; McClelland & O’Regan, 1981a, 1981b; Rayner & Slowiaczek, 1981, see also Vasilev & Angele, 2017).

The third objective of the study was to explore the neural correlates of the foveal lexical frequency effect under natural reading conditions. Based on previous research, we expected to replicate word frequency effects in the eye movement data. However, given that the neural correlates of word frequency have rarely been investigated during natural reading, we were less confident in our predictions for frequency effects in the FRP data. Recall that Kretzschmar et al. (2015) investigated frequency effects using coregistration during natural reading and failed to obtain reliable correlates of eye movement effects. Thus, if we obtained such effects in our study, it would be the first demonstration of these effects in a natural reading task. Alternatively, a failure to obtain such effects would be consistent with Kretzschmar et al. (2015), but would leave us with the difficult job of explaining quite why the effects did not occur. To ensure that we were comprehensive in our attempts to identify any such effects, we explored processing early after fixation onset (i.e., before 300 ms), as well as later during processing (i.e., between 300 ms and 500 ms) across a broad scalp distribution.

## Method

### Participants

Forty-two participants (30 female) took part in the study. All participants were English native speakers, right-handed (*M* = 85.38, *SD* = 17.05 according to the Edinburgh Handedness Inventory; Oldfield, 1971), with normal or corrected-to-normal vision, no reading disabilities, and no history of neurological disorders. Participants’ age ranged between 18 and 26 years (*M* = 19.29, *SD* = 1.60). All participants provided written informed consent and received course credits or money for taking part.

### Stimuli

We selected 696 five- or six-character words from the English Lexicon Project (Balota et al., 2007), 348 high-frequency words (between 101.12–5863.88 frequency per million) and 348 low-frequency words (between 5.05–24.80 frequency per million). We matched pairs of high- and low-frequency targets, in order to create 174 sentence frames, each containing two high-frequency or two low-frequency target words. Twenty participants from the University of Southampton assessed these sentences for plausibility, rating each sentence on a scale from 1 = *very implausible* and 7 = *very plausible*. Another group of 30 participants took part in a cloze predictability task, completing the sentence fragment up to, but not including, each target word, with the first word that came to mind. From the norming tests, we chose 108 sentences (*M* = 75.74, *SD* = 4.23 characters long) that did not differ significantly in terms of plausibility (high-frequency [HF] target words between 3.20–6.30, low-frequency [LF] target words between 3.20–6.11; *p* = 0.17) and predictability (between 0.00–0.20 for both HF and LF target words; *p* = 0.52). The characteristics of the set of words used in the experiment are provided in Table 1.[Table-anchor tbl1]

### Design

We used a 3 (Parafoveal Preview: X string preview vs. letter string preview vs. identical preview) × 2 (Target Word Frequency: high vs. low frequency) experimental design, with 18 sentences and 36 target words per condition. Sentences were presented using the boundary paradigm (Rayner, 1975). As illustrated in Figure 1, two invisible boundaries were located within each sentence, before the space preceding the target words. Before crossing each of the two boundaries, the target word was replaced with a parafoveal preview. The preview could be comprised of Xs, or strings of random letters that were visually similar to the letters of the original word but which did not carry any meaning (and were orthographically illegal). In the final condition, the preview was identical to the target word. Once the participants’ eyes crossed the boundary, the preview was replaced by the correct target word, which was either a high-frequency (left panel of Figure 1) or low-frequency target word (right panel of Figure 1).

### Apparatus

Participants were seated 70 cm from a 19-in. stimulus CRT display screen, with a resolution of 1,024 × 768 and a refresh rate of 140 Hz. The text was presented in 14-point Courier New sentence case font, in black ink on a gray background. Approximately 2.19 characters subtended one degree of visual angle.

Viewing was binocular, but eye movements from the right eye only were recorded from a desktop EyeLink 1000 eye tracking system (SR Research), at a sampling rate of 1000 Hz. A 3-point calibration procedure was completed at the beginning of each block of sentences, and whenever needed during the experiment. Calibration was accepted when the average error was lower than 0.3° and the maximum error lower than 0.99°. Furthermore, a 2-point drift correction check was performed at the beginning of each trial.

The EEG signal was recorded from 64 scalp electrodes (Fast’n Easy Cap, Herrsching, Germany) located according to the 10–20 International system. Four EOG channels were also used to record the EEG signal associated with eye movements. AFz was used as ground electrode, and the nose as the online reference. The EEG signal was recorded from DC SynAmpsRT amplifiers (Compumedics Neuroscan, Charlotte, NC) with a sampling rate of 1000 Hz, and it was low-pass filtered online at 100 Hz (with an attenuation of 12dB/octave).

### Procedure

The current experiment was approved by the University of Southampton Ethics Committee (study ID: 25066).

Before starting the experiment, participants were required to complete the Edinburgh handedness inventory (Oldfield, 1971) in order to check that all participants were right-handed. Next, participants were tested for their visual acuity and required to meet 20/20 vision on the Landolt “C” eye chart (Precision Vision, La Salle, IL), at 4-m viewing distance. Lastly, a calibration procedure was performed to ensure that participants’ eyes could be accurately tracked. Participants who successfully completed the above procedures, continued with the actual experiment.

The experimental session involved five blocks of sentences. The first block always comprised 10 practice trials to familiarize the participant with the procedure. The following four blocks were formed from one filler as the first trial and 27 experimental sentences, which were presented in a random order for each participant. Participants were asked to silently read each sentence and to answer comprehension questions for 24% of the trials, while their eye movements and EEG signal were simultaneously recorded.

Each trial began with a cross on the left side of the screen. Participants were required to fixate the cross for 500 ms before a sentence was displayed, with the first letter of the sentence located at the same position of the cross. Once participants had silently read the sentence to understand it to the best of their ability, they were instructed to fixate a cross on the right side of the screen to terminate the current trial and initiate the following one.

After the experiment, participants were asked to complete a short questionnaire to assess their display change awareness (Dimigen et al., 2012; White, Rayner, & Liversedge, 2005). The experimental session lasted about 1 hr, with the opportunity for the participants to have breaks at the end of each block of sentences or at any point if needed.

### Coregistration of Eye Movements and EEG Signal

At the onset and offset of each trial the stimulus display computer (running SR Research Experiment Builder) sent a trigger to the computer recording the EEG signal and a message to the computer registering eye movements. This allowed for an accurate offline synchronization of eye movements and EEG signal via the EYE-EEG extension of EEGLAB toolbox (Dimigen et al., 2011), as confirmed by a correlation of 1 between the markers of both recordings, and deviations equal or shorter than 1 ms in absolute value (*M* = 0.29, *SD* = 0.46 ms in absolute value).

### Preprocessing of Eye Movement Data

The eye movement data were pruned via the “clean” function in DataViewer (SR Research) such that only fixations longer than 50 ms and shorter than 800 ms entered the analyses. Fixations on each interest area were excluded when the display change occurred early, during a fixation on the preboundary (i.e., pretarget) word, and when the display change was late (i.e., when the display change took more than 10 ms after fixation onset on the target). We also removed fixations with hooks, wherein the display change was triggered early by a saccade that temporarily crossed the invisible boundary to finally end to the left of the boundary. Furthermore, we excluded from the analyses fixations on pretarget and target words in which participants made a blink and/or a skip. Additionally, only consecutive fixations, which landed first on the pretarget word and then proceeded onto the target word, entered the analyses. Lastly, only fixations that occurred during first-pass reading were analyzed.

### Preprocessing of FRP Data

**Figure 2 fig2:**
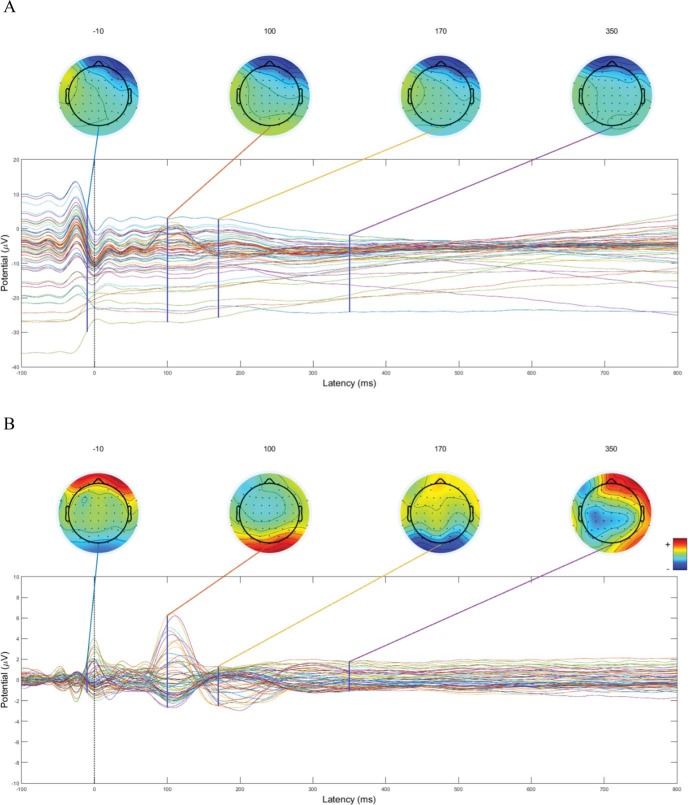
Results from the FRPs preprocessing and artifact correction procedure. Scalp topographies are shown for specific time points to account for muscle spike potential (−10 ms), P1 (100 ms), N1 (170 ms), and N400 (350 ms) components for raw (panel A) and artifact corrected (panel B) grand averages of FRPs time-locked to the fixation onset of the pretarget and target words.

The EEG data were band-pass filtered offline with the EEGLAB 14_1_1 (Delorme & Makeig, 2004) toolbox for Matlab (version R2015a), between 0.1 Hz and 30 Hz. Independent component analysis (ICA) was performed in order to identify the ocular artifacts. To optimize the ICA decomposition, the extended Infomax ICA algorithm (Bell & Sejnowski, 1995; Lee, Girolami, & Sejnowski, 1999; Makeig, Bell, Jung, & Sejnowski, 1996) was trained on specific segments of the EEG signal (e.g., Debener, Thorne, Schneider, & Viola, 2010; Meyberg, Sommer, & Dimigen, 2017). These segments were time-locked to fixation onsets and comprised 0.1 s before and 0.5 s after fixation onset (i.e., a sufficiently long epoch to include previous and following saccades). Before segmenting to training set, the data were high-pass filtered at 1 Hz to reduce slow and unsteady drifts that are spatially nonstationary through time (for discussion about ICA assumptions and caveats, see Onton, Westerfield, Townsend, & Makeig, 2006). Next, the ICA weights were applied to the band-pass filtered (i.e., 0.1 Hz–30 Hz) data, the EEG signal was segmented into epochs of 900 ms cut around each fixation onset (−100 ms to +800 ms), and the independent components associated with ocular artifacts identified according to the EYE-EEG extension (Dimigen et al., 2011). The independent components that shared temporal covariance higher than 1.1 with eye movements were pruned from the data (*M* = 2.98 components removed per participant, *SD* = 1.52) as oculomotor artifacts (Plöchl, Ossandón, & König, 2012; see Figure 2). The artifact-free EEG segments were then rereferenced against the mean of all scalp electrodes (average reference) and baseline-corrected by subtracting the 100 ms preceding the fixation onset on the pretarget word, regardless of the epochs time-locked to the fixation onset of pretarget or target word. The choice of this baseline is founded on the rationale that when we read we extract information from the parafovea (Rayner, 1998, 2009). Therefore, the time window prior to the fixation onset on the target word may be affected by the characteristics of the parafoveal information that is being extracted, and as a consequence, this may bias the results that are to be observed on the FRPs time-locked to the fixation onset of the target word. To exclude nonocular artifacts, segments with a peak-to-peak voltage difference greater than 120 μV (in absolute value) in any scalp channel were rejected (*M* = 5.38 segments removed per participant, *SD* = 8.16). Spherical interpolation of a channel was performed when that channel exceeded the threshold for more than 5% of all epochs, which was the case for nine of our participants. FRPs were then averaged within and then across participants for analyses. After eye movement and FRP preprocessing, we were left with a total number of 5,349 observations for the pretarget and target word.[Fig-anchor fig2]

### Eye Movement Statistical Analyses

Eye movement data were analyzed with linear mixed effects models within the R environment for statistical computing (R Core Team, 2015). We used the “lmer” function from the lme4 package (Bates, Mächler, Bolker, & Walker, 2015) on log transformed first fixation duration (i.e., the duration of the first fixation on a region during reading), single fixation duration (i.e., the duration of the fixation on a region when the reader only made one fixation on it during first pass reading), and gaze duration (i.e., the sum of all first-pass fixations on a region, before readers fixate another region).

Word frequency and parafoveal preview type were coded as fixed factors and specified using the function “contr.sdif” from the MASS package (Venables & Ripley, 2002). Given that the factor parafoveal preview type included three levels (identical vs. string of random letters vs. string of Xs), we used the function “relevel” to perform those additional a priori contrasts that we could not attain with the original model. Initially, the full random structure, with both random intercepts and slopes, was included for both subjects and items as per Barr, Levy, Scheepers, and Tily (2013). All final models reached convergence with a full random structure for subjects, but not for items. When the models failed to converge, we systematically reduced the random structure by first trimming down the items level correlation. If a model still did not converge, we reran the model excluding the interaction between word frequency and parafoveal preview type in the random structure. If the model still failed to reach convergence, both the correlation and interaction were removed, and if still unsuccessful, each random slope was removed one-by-one (first removing word frequency). The *p* values were estimated using the “lmerTest” package, with the default Satterthwaites’s method for degrees of freedom and t-statistics (Kuznetsova, Brockhoff, & Christensen, 2017).

### FRP Statistical Analyses

To investigate the neural correlates of visual, orthographic, and lexical PoF effects, we analyzed FRP epochs time-locked to the fixation onset of the pretarget word. Orthographic PoF effects were previously found in semantic relatedness decision tasks with pairs of prime-target words around 119 ms at left occipital sites and around 140 ms at right central and frontal electrodes (Baccino & Manunta, 2005), and between 200 ms and 280 ms at occipital sites (Simola, Holmqvist, & Lindgren, 2009). However, no previous study has investigated FRP amplitudes associated with the processing of a word *n* (pretarget word) as a function of the visual and orthographic properties of the upcoming parafoveal word *n* + 1 (target word) during natural reading. With respect to PoF effects of lexical frequency, only one existing study has found evidence of these effects (Niefind & Dimigen, 2016). They observed significant differences between 130 ms and 140 ms at frontal electrodes (AF8 and F4), and between 630 ms and 640 ms at one centro-parietal electrode (CP5). However, the study involved saccadic reading of word lists, with the authors acknowledging that later effects may have been delayed due to the less natural reading paradigm. Therefore, we decided to analyze FRP mean amplitudes time-locked to the fixation onset of the pretarget word over three time windows, that is, between 70 ms and 120 ms, 120 ms–300 ms and 300 ms–500 ms. These time-windows were chosen to investigate early components that are known to show effects of visual and orthographic manipulations, that is, P1 and N1 components, and a later component that typically shows effects of lexical frequency, that is, the N400 component. To detect reliable differences between conditions, the FRP mean amplitudes of these conditions were submitted to a two-tailed nonparametric cluster-based permutation test (Maris & Oostenveld, 2007) using the Matlab toolbox *FieldTrip* (Oostenveld, Fries, Maris, & Schoffelen, 2011). All scalp electrodes and all time points of interest for each time window were included in the test. We generated the permuted data with 5,000 iterations by randomly assigning the condition labels of each participant’s response averages on each iteration. Then, for each iteration, we computed a dependent samples *t*-statistic for each sample (channel-time pair) for the difference between the conditions. If the *t*-statistic was smaller than a threshold of 0.05 and was temporally and spatially adjacent^2^ to another point with a significant *t* value, we assigned this *t* value to a cluster. For each iteration, we then computed the maximum (in absolute value) positive and negative cluster-level *t*-statistic by calculating the sum of all the *t* values within a cluster and we created the null distribution. For the original (observed) data, we applied the same procedure except that we did not shuffle the trials between conditions. The observed *t*-statistic was tested against the null distribution of the permuted data. The observed cluster-level *t*-statistic was considered significant (with a *p* value less than .025 in each tail) when it was located beyond the determined threshold (i.e., the 5% of the most extreme maximum/minimum cluster-level *t*-statistic over the null distribution). This type of permutation test is a mass univariate approach that allows for a considerable number of univariate tests (e.g., *t* tests) to be performed to compare the electrical activity of different conditions at each sample, that is at each of the multiple scalp locations and at each of the multiple time points, while controlling for the multiple comparisons problem. Indeed, the assumption is that real effects will typically occur over multiple temporally and/or spatially adjacent time points and electrodes (Groppe, Urbach, & Kutas, 2011b). Compared with other methods, the advantage of the cluster-based approach is to provide a good spatial and temporal resolution of the effects, with a strong degree of certainty in detecting the presence of an effect, yet controlling for the large number of comparisons performed (Groppe, Urbach, & Kutas, 2011a; Maris & Oostenveld, 2007).

To examine the neural correlates of visual and orthographic preview effects and foveal effects of lexical frequency, we analyzed the FRP epochs time-locked to the fixation onset of the target word. Based on the existing studies with saccadic reading and RSVP-with-flanker-word-presentation, we expected to find similar effects of preview validity on the occipito-temporal electrodes between 140 ms and 200 ms (Niefind & Dimigen, 2016) and between 200 ms and 300 ms (Dimigen et al., 2012; Kornrumpf et al., 2016; Li et al., 2015) after fixation onset, and effects at the middle-central electrodes between 300 ms and 500 ms (Dimigen et al., 2012; Kornrumpf et al., 2016; Li et al., 2015).With respect to the lexical frequency of the target word, previous studies using a RSVP paradigm reported significant effects in early and late time windows (between 100 ms and 500 ms after stimulus onset) across different electrode sites (fronto-central, centro-parietal and occipital areas; see Laszlo & Federmeier, 2014 for a review). In those studies, that have used more natural reading paradigms, such as RSVP-with-flanker-word-presentation and saccadic reading, effects of target frequency have been observed at occipito-temporal and fronto-central electrodes between 200 ms and 300 ms, and between 140 ms and 300 ms, respectively (Niefind & Dimigen, 2016). However, in the single coregistration study that explored the lexical frequency during natural reading (Kretzschmar et al., 2015), no significant effect was observed between 150 ms and 650 ms after fixation onset at centro-parietal electrode sites.

Because neural correlates of preview effects have not yet been established under natural reading conditions, and because the frequency effects studied with reading of sentences have been examined only over specific areas of the scalp, our aim was to provide a more comprehensive picture of such effects for normal reading. Thus, we analyzed the FRP mean amplitudes time-locked to the fixation onset of the target word over four time windows, that is, between 0 ms and 70 ms, 70 ms–120 ms, 120 ms–300 ms and 300 ms–500 ms on a millisecond by millisecond basis at all scalp electrodes using the same nonparametric cluster-based permutation statistics (Maris & Oostenveld, 2007) described above. As for the pretarget word FRP analyses, the same time windows were locked to the target fixation onset to investigate early (i.e., P1 and N1) and later (i.e., N400) components. We included an additional early time-window, between 0 ms and 70 ms, because we anticipated that preview information would be extracted from the target region prior to its direct fixation and this information could have a relatively immediate impact on processing of the target after the eyes had crossed the boundary.

## Results and Discussion

### Behavioral Data

#### Accuracy

The average accuracy for comprehension questions was 93% (*SD* = 4.12), showing that participants read and understood the sentences.

#### Display change awareness

Out of the 42 participants, 37 participants reported having noticed that something unusual occurred on the display (33 participants reported it spontaneously, and four participants after being informed of the changes). Participants estimated on average that 32 display changes (*SD* = 21.01) occurred (in fact 72 display changes actually occurred in the experimental trials). Thirty-two of the aware participants perceived that one or more words in the sentence were replaced by string of Xs, while nine participants noticed that one or more words were replaced by jumbled letters. This finding is consistent with a number of studies in the literature (Angele et al., 2016; Slattery et al., 2011) reporting that participants are able to detect changes when presented with a preview that differs from the target, and that sensitivity to a display change is increased if the preview is less word-like (e.g., string of Xs) compared with more word-like (e.g., string of letters).

### Parafoveal-on-Foveal Effects

To examine the influence that parafoveal processing of words to the right of fixation can have on the processing of the foveal word, we analyzed eye movements and FRPs time-locked to the fixation onset on the pretarget word. Given that all the words preceding and following the target were the same in all conditions, at the pretarget word any difference that we observe must be caused by the parafoveal information (i.e., the preview or frequency of the target word).

#### Visual and orthographic PoF effects

One of the goals of the current study was to investigate the neural correlates of visual and orthographic PoF effects during natural reading, as to date, these effects have only been studied in relation to paradigms presenting word pairs (Baccino & Manunta, 2005; Simola et al., 2009) or lists of words (Dimigen et al., 2012).

The eye movement data showed that reading times on the pretarget word were longer when the parafoveal preview was formed from Xs compared with when the preview was the target word itself or comprised of a string of random letters (see Table 2). The difference between X-string preview and identity preview conditions was significant at the pretarget word for both single fixation duration (difference of 8 ms) and gaze duration (difference of 21 ms) and marginally significant on first fixation duration (7 ms), while the difference between X-string previews and letter-string previews reached significance on gaze duration (difference of 17 ms) and was marginally significant on first fixation duration (difference of 8 ms; see Table 3). Interestingly, we did not observe any significant difference on any measure at the pretarget word for letter-string previews compared with identity previews.[Table-anchor tbl2][Table-anchor tbl3]

The eye movement results indicate that participants were quite sensitive to visually unusual strings in the parafovea (i.e., string of Xs), but they were less sensitive to visually word-like stimuli (i.e., visually similar orthographically illegal strings of letters). These findings appear to be consistent with previous evidence that readers obtain at least visual information from words in the parafovea before fixating it (e.g., McConkie & Rayner, 1975; Morris, Rayner, & Pollatsek, 1990), and replicate aforementioned studies that did not find orthographic PoF effects (e.g., White & Liversedge, 2004, 2006; though see Angele, Slattery, Yang, Kliegl, & Rayner, 2008; Inhoff et al., 2000; Rayner, 1975).

**Figure 3 fig3:**
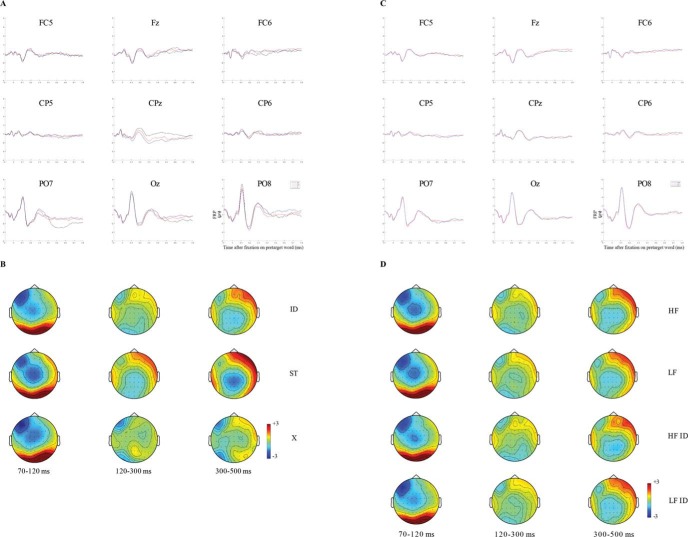
Results from FRPs time-locked to the fixation onset on the pretarget word. Panel A: Grand average FRPs in response to ID (i.e., identical preview), ST (i.e., random letter string preview), and X (i.e., X-string preview) conditions displayed on nine channel locations: left frontal (FC5), midline frontal (Fz), right frontal (FC6), left centro-parietal (CP5), midline centro-parietal (CPz), right centro-parietal (CP6), left parieto-occipital (PO7), midline occipital (Oz), and right parieto-occipital (PO8) electrodes. Panel B: Topographies showing the average brain activity associated with ID, ST, and X conditions for three time windows: between 70 ms and 120 ms, between 120 ms and 300 ms, between 300 ms and 500 ms after fixation onset on the pretarget word. Panel C: Grand average FRPs in response to HF (i.e., high-frequency) and LF (i.e., low-frequency) conditions displayed on the same nine channel locations as in panel A. Panel D: Topographies showing the average brain activity associated with HF, LF, HF ID (i.e., high-frequency target word with identical preview), and LF ID (i.e., low-frequency target word with identical preview) conditions for three time windows: between 70 ms and 120 ms, between 120 ms and 300 ms, between 300 ms and 500 ms after fixation onset on the pretarget word.

Next, let us consider the FRP data time-locked to the pretarget word. The topographies representing the FRP grand averages are shown in Figure 3. The first thing to note is that all conditions elicited a robust P1 component, as can be seen by the positive brain activation over the posterior areas of the scalp.^3^ The latency of this P1 component is thought to reflect visual encoding (see Dien, 2009 for a review). This activation dissipated quite rapidly within the following window between 120 ms and 300 ms and developed into more negative brain activity over the left occipital and central areas of the scalp, providing evidence of a N1 component for all conditions (note, though, there are clearly differences in the intensity and scalp locations of this effect across conditions). Furthermore, in the next successive time window, that is between 300 ms and 500 ms, some evidence of a N400 component in the central sites of the scalp was observed in the identity and letter-string preview conditions, but not for the X-string preview condition. We consider that this differential state of activation indicates that little useful processing of the parafoveal stimulus was possible for X-string previews, whereas preprocessing of the parafoveal stimulus could occur for letter-string and identity preview conditions.[Fig-anchor fig3]

**Figure 4 fig4:**
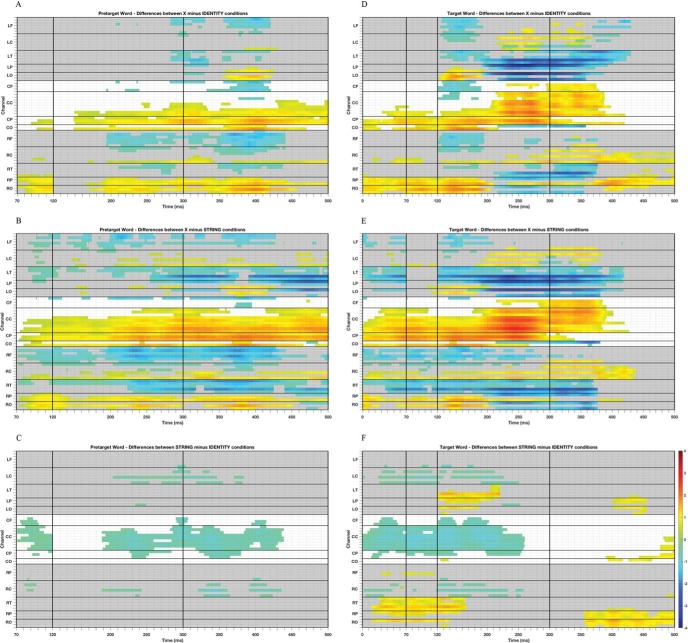
Raster diagrams illustrating significant FRP differences obtained with cluster-based permutation tests. Red and blue rectangles indicate channel/time point in which the first condition is significantly more positive or negative of the second condition, respectively. Channels are displayed on the *y*-axis and organized somewhat topographically. Channels on the left hemisphere (L) of the scalp are shown on the figure’s top gray rectangle and demarcated with horizontal lines based on their location on the frontal (LF: FP1, AF7, AF3, F7, F5, F3), central (LC: FC5, FC3, C5, C3, CP5, CP3), temporal (LT: FT9, FT7, T7, TP9, TP7), parietal (LP: P7, P5, P3), and occipital (LO: PO7, PO3, O1) regions of the scalp. Midline electrodes (C; i.e., CF: FPz, F1, Fz, F2; CC: FC1, FCz, FC2, C1, Cz, C2, CP1, CPz, CP2; CP: P1, Pz, P2; CO: Oz, POz) are displayed in the middle. Right channels (R) are shown on the figure’s bottom gray rectangle (i.e., RF: FP2, AF8, AF4, F8, F6, F4; RC: FC6, FC4, C6, C4, CP6, CP4; reaction time [RT]: FT10, FT8, T8, TP10, TP8; RP: P8, P6, P4; RO: PO8, PO4, O2). The time from the onset of a fixation on the pretarget (panels A, B, and C) and target (panels D, E, and F) words is displayed on the *x*-axis. The vertical black lines indicate the time windows considered for the cluster-based permutation tests: between 70 ms–120 ms, 120 ms–300 ms, 300 ms–500 ms for analyses of pretarget words, and between 0 ms–70 ms, 70 ms–120 ms, 120 ms–300 ms, 300 ms–500 ms for analyses of target words. Panels A: FRP differences between pretarget words with previews made of Xs and identity previews; B: between previews made of Xs and previews made of string of letters; C: between previews made of string of letters and identity previews. Panels D: FRP differences between target words with previews made of Xs and identity previews; E: between previews made of Xs and previews made of string of letters; F: between previews made of string of letters and identity previews.

The left panels of Figure 4 show the significant voltage differences between each pair of the three preview conditions found with the cluster-based permutation tests (see Table 4 for a summary of these differences). Each of the panels presents a large amount of information, and given this, it is important to focus on those aspects that reflect the most substantive differences in processing that exist across conditions. With this in mind, it is evident that the greatest number of significant differences is observed when data from the conditions in which previews were comprised of Xs are compared with the conditions in which previews were identical to the target word (7,775 differences; see Figure 4A), and when the previews were strings of random letters (14,404 differences; see Figure 4B). The least number of differences is observed when comparing previews comprised of strings of random letters with previews identical to the target word (3,017 differences; see Figure 4C). Without question, the identity and letter-string previews are the most visually similar, and both are strikingly visually dissimilar to X-string previews. It is, therefore, unsurprising that the most substantive differences in activation patterns that can be seen across the left panels of Figure 4 for the pretarget word reflect these particular contrasts.[Fig-anchor fig4][Table-anchor tbl4]

Given that this study provides the opportunity to investigate whether different types of preview have a different influence on parafoveal processing, let us focus our attention on Figure 4A and Figure 4C. These panels convey the differences in processing associated with parafoveal X-strings relative to the identity string, and parafoveal random letter strings relative to the identity string. Dealing first with Figure 4A, we can see that the earliest positive difference in activation occurs over the right and midline parietal and occipital areas and is sustained throughout the entire temporal window of analysis. A slightly later effect, mainly rightward, can be seen to develop over frontal and temporal areas, and over time, this effect spreads more bilaterally before dissipating approximately 430 ms–440 ms after fixation onset. With respect to the spatial characteristics of these data, the involvement of the right hemisphere (RH) is in line with the growing body of evidence indicating that the RH plays an important role in the extraction and processing of the visual form of words (e.g., Deason & Marsolek, 2005; Lindell, 2006), as well as processing of unpronounceable orthographically irregular items (e.g., Dickson & Federmeier, 2014). Furthermore, the early significant differences observed over parietal and occipital regions and the subsequent later activation over temporal and frontal areas of the scalp are consistent with previous work showing that word-related activation progresses from posterior to anterior regions (e.g., Friederici, 2011; Marinkovic et al., 2003; Shtyrov & MacGregor, 2016). Considering now Figure 4C, there are several noteworthy points. First, the differences that can be seen in this panel are small (though significant) and all occur at central locations. Activation that occurs in these regions during processing of words is ordinarily associated with cognitive processes beyond visual encoding of stimuli and processing of isolated letters (e.g., Carreiras, Armstrong, Perea, & Frost, 2014). Another noteworthy point is that our results partially replicate those of Simola et al. (2009) and Baccino and Manunta (2005). Simola et al. (2009) found a negative difference between illegal letter combinations and real words (i.e., both for semantically related and unrelated words) that occurred between 200 ms and 280 ms. Thus, there was similarity between Simola et al.’s (2009) results and the present results in relation to temporal course. Baccino and Manunta (2005) obtained a negative difference between illegal letter combinations and unrelated target words at the right central and frontal electrodes. Thus, these results were comparable in relation with the scalp distribution of the effects.

In sum the FRP results confirm our predictions that processing letter strings in the parafovea is far more similar to processing words in the parafovea than X-strings. Random letter strings appear word-like in the parafovea, thus, readers engage in visual and orthographic processing. However, some modest disruption occurs, likely during orthographic, or even morphological processing of a parafoveal letter string. In contrast, parafoveal previews comprised of Xs share very few features or letters (if any) with words. Readers are sensitive to this visual oddity in the parafovea, and upon detection of a string that is so unlike a word, they desist from continued (normal) parafoveal processing of it. We believe that it is for this reason that we observe the greatest disruption to processing at the pretarget word for this condition.

#### Lexical parafoveal-on-foveal effects

As we have noted, previous studies investigating lexical PoF effects have been inconsistent both in the eye movement and in the FRP literature. Niefind and Dimigen (2016) observed PoF effects of word frequency between 130 ms and 140 ms at right frontal electrodes, and between 630 ms and 640 ms at left parietal electrodes, in a saccadic word-list reading task. Kretzschmar et al. (2015) analyzed a time window between 150 ms and 400 ms after fixation onset over centro-parietal sites and did not observe lexical PoF effects in a natural reading paradigm. Thus, our study aimed to understand whether the different results could be due to the choice of time windows and brain areas investigated, or the type of paradigm used. To address the first hypothesis, therefore, we decided to consider all scalp electrodes and a wider range of latencies compared with Kretzschmar et al. (2015). Let us turn next to the second hypothesis, namely, that the paradigm may be critical in determining the nature of effects. When words are embedded in meaningful sentences, reading times are substantially shorter on average than when they are embedded in lists of words. The reason for this is very likely that when readers process lists of individual words, they cannot engage the natural language comprehension processes that occur quite automatically during natural reading. To be clear, word list reading is an artificial task that does not lend itself naturally to the rapid automatized processes that occur spontaneously when a person reads for comprehension. Thus, the artificiality of the task leads to staccato processing of words, which in turn causes a more stilted reading style relative to natural sentence reading. In short, the time course of processing for each word will very likely be slower. If this suggestion is correct, then FRPs recorded during natural reading of sentences might be less sensitive to lexical PoF effects because (a) the time course of processing is faster and there is insufficient opportunity for the extraction of lexical information from the parafovea, and (b) the magnitude of any lexical PoF effects may be reduced because fixations are truncated earlier in the former than the latter situation resulting in far weaker effects.

Analyses of eye movement measures on the pretarget word did not reveal any significant main effect of target word frequency, nor an interaction between parafoveal preview and target word frequency (see Table 3). These results were confirmed by the FRP data. Comparisons between high- and low-frequency conditions did not reveal any significant clusters in either of the analyzed time windows. We also checked the identity conditions alone to assess whether there was any suggestion of a PoF effect for the high- and low-frequency target words, and there was none. Additionally, even though we explored a wider range of brain areas and latencies than Kretzschmar et al. (2015), we failed to find any evidence for extraction of lexical information from the parafovea. Our results here provide no evidence for PoF effects in any brain region for any time window in our analyses.^4^ Furthermore, our results do not offer a clear explanation for the contradictory nature of the results obtained by Niefind and Dimigen (2016) and Kretzschmar et al. (2015). It remains possible that ours and Kretzschmar et al.’s (2015) failure to obtain such effects occurred because fixations were shorter during natural reading than during word-list reading, however, what we can say with some confidence is that Kretzschmar et al. (2015) did not fail to detect PoF effects because they examined inappropriate time windows and brain areas. Further studies are needed to determine whether sentence reading paradigms do not elicit PoF effects of frequency at all, or whether they elicit a much weaker and short-lived response to parafoveal word frequency.

Finally, if readers process words in parallel, then we expected that they should be sensitive to orthographically illegal letter strings in the parafovea that do not have lexical status. As such, we would expect very large and significant differences in activation between the identity and letter string condition at the pretarget word (see Figure 4C). Our failure to obtain such effects is consistent with the suggestion that readers do not process words in parallel (at least to the extent that they ascertain the lexical status of a parafoveal string). The current results are in line with other studies that have failed to obtain robust lexical PoF effects in reading (e.g., Brothers et al., 2017).

### Visual and Orthographic Preview Effects

One of the aims of our study was to see whether we could replicate in natural reading the findings for neural correlates that have been observed in RSVP-with-flanker-word presentation and saccadic word list reading experiments. We were particularly keen to explore for the first time whether differences in neural correlates could be observed at a target word that was preceded by a preview of a string of random letters compared with a string of Xs. Recall that in previous studies (Dimigen et al., 2012; Kornrumpf et al., 2016; Li et al., 2015; Niefind & Dimigen, 2016), an early *preview positivity* effect was obtained such that there was an attenuation (reduced negativity) of the N1 component, as well as a later effect such that there was an attenuation of the N400 component, when a word was preceded by an identical preview relative to several forms of invalid preview. Therefore, at the target word, we predicted differences in those component latency ranges between the identity and the X-string preview conditions, and between the identity and letter string preview conditions. In relation to the former comparison, we might suggest that X-string previews would delay any form of effective orthographic processing of the target (relative to processing in the identity condition) because these previews are so very visually and orthographically different from the target. In relation to the random letter string preview condition, we were more cautious, because to date there has been no work to examine how different forms of preview impact on neural correlates of lexical processing. Somewhat tentatively, we might expect that a letter string preview would produce inhibitory effects due to the dissimilarity of preview and target, but to a smaller extent than X-string previews, due to the fact that the letter string previews were constructed using visually similar letters to the target, and therefore, they share common features.

First, note that analyses of the eye movement data replicated the well-established preview effect. For all our eye movement measures (see Table 2 and Table 3), fixation durations on the target word were significantly shorter when participants received a valid preview in the parafovea (i.e., the target word itself) compared with when they received an invalid preview comprised of random letters (difference of 41 ms in first fixation duration, 57 ms in single fixation duration, 58 ms in gaze duration) or comprised of Xs (difference of 41 ms in first fixation duration, 63 ms in single fixation duration, 61 ms in gaze duration). Note that the magnitude of the preview benefit effect was comparable regardless of whether the preview was formed from random letters or an X-string (cf., Vasilev & Angele, 2017).

**Figure 5 fig5:**
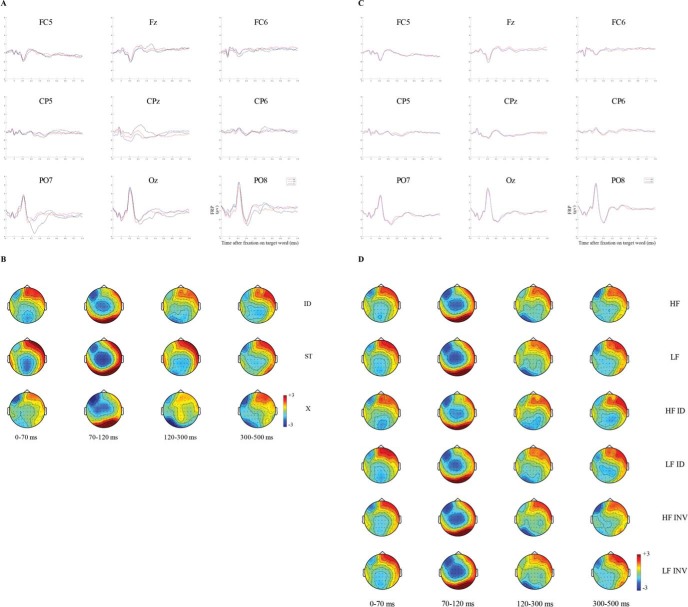
Results from FRPs time-locked to the fixation onset on the target word. Panel A: Grand average FRPs in response to ID (i.e., identical preview), ST (i.e., random letter string preview), and X (i.e., X-string preview) conditions displayed on nine channel locations: left frontal (FC5), midline frontal (Fz), right frontal (FC6), left centro-parietal (CP5), midline centro-parietal (CPz), right centro-parietal (CP6), left parieto-occipital (PO7), midline occipital (Oz), right parieto-occipital, and (PO8) electrodes. Panel B: Topographies showing the average brain activity associated with ID, ST, and X conditions for four time windows: between 0 ms–70 ms, 70 ms–120 ms, 120 ms–300 ms, 300 ms–500 ms after fixation onset on the target word. Panel C: Grand average FRPs in response to HF (i.e., high-frequency) and LF (i.e., low-frequency) conditions displayed on the same nine channel locations as in panel A. Panel D: topographies showing the average brain activity associated with HF, LF, HF ID (i.e., high-frequency target word with identical preview), LF ID (i.e., low-frequency target word with identical preview), HF INV (i.e., high-frequency target word with invalid preview, that is both X-string and string of random letters), LF INV (i.e., low-frequency target word with invalid preview, that is both X-string and string of random letters) conditions for four time windows: between 0 ms–70 ms, 70 ms–120 ms, 120 ms–300 ms, 300 ms–500 ms after fixation onset on the target word.

Next, let us consider the FRP grand averages shown in Figure 5. In this figure, notice that we have an additional time window for our FRP analyses, namely, that between 0 and 70 ms. We analyzed FRPs in this window as here one might anticipate effects that reflect processing associated with the preview that have spilled over and occur in the earliest period of the fixation on the target word. Indeed, for the identity and random letter string conditions at this time window, there is some suggestion of some centralized negativity that may reflect processing of the preview that remains at fixation onset on the target. There is no suggestion of such an effect for the X-string preview condition, and this is in line with our earlier suggestion that parafoveal processing cannot progress effectively for X-string previews. Next, for the 70 ms–120 ms window, in all three preview conditions we can see a pronounced P1 component such that positive activation at posterior sites is quite apparent. Also, the component is most pronounced in the letter string preview condition, and we speculate that this might be so due to conflicting influences of the visually similar preview. If correct, the implication here is that readers initiated orthographic processing of the preview prior to fixation of the target. Finally, let us consider the remaining time windows together, first for the X-string preview condition, and then for the letter string and identity preview conditions. For the X-string preview condition, we can see a clear N1 component over the left temporal and occipital areas between 120 ms and 300 ms which is then sustained into the following time window. Consistent with several studies (e.g., Dimigen et al., 2012; Niefind & Dimigen, 2016), this component very likely reflects orthographic processing of the target that became available from fixation onset. For the identity and letter string preview conditions, we can also see a N1 component particularly so in the 120-ms to 300-ms time window; however, here it is much less pronounced presumably because orthographic processing was initiated earlier in these conditions relative to the X-string conditions. This effect is slightly dissipated and shifts to a more central location between 300 ms and 500 ms.[Fig-anchor fig5]

Let us now turn to the permutation analyses (see Table 4 for a summary of the differences) illustrated in the raster diagrams representing the differences between preview conditions at the target word (Figure 4D–4F). As per the figures for the pretarget word, there were a greater number of significant voltage differences when comparisons were made between the X-string preview conditions and the identity condition (12,012 differences; see Figure 4D), and the X-string preview conditions and the letter string preview condition (14,452 differences; see Figure 4E). The least number of significant voltage differences was observed when comparisons were made between letter string preview condition and the identity condition (6,627 differences; see Figure 4F). Again, given that X-string previews are markedly visually different from random letter string and identity previews, it is unsurprising that there are correspondingly increased differences in neural correlates across Figures 4D–4F. Given that one of our objectives was to investigate whether differences in neural correlates could be observed when the target was preceded by a preview of a string of random letters compared with a string of Xs, we will focus on Figure 4D and Figure 4F. Figure 4D represents the significant voltage differences between the X-string and the identity previews. The first point to note here are the differences in the right and midline parietal and occipital areas during the two earliest time windows (0 ms–70 ms and 70 ms–120 ms). These differences likely indicate that in the X-string preview condition, it is only at fixation onset, once the target has replaced the preview, that visual processing of the form of the target itself is initiated. In contrast, in the identity preview condition, such processing was initiated earlier (when the word was in the parafovea). Thus, the differences are apparent from fixation onset and are sustained throughout the majority of the time windows we considered. These results are consistent with previous evidence showing that early components, for example P1, are associated with analysis of visual features (see Dien, 2009 for a review), over low-level perceptual areas in the posterior regions of the brain (e.g., Heinze et al., 1994; Martínez et al., 1999). From Figure 4D we can also see very clearly that from the start of the time window spanning from 120 ms–300 ms there are significant activation differences across a range of areas (temporal, central, and frontal) that all have an onset close to 120 ms.

In line with previous studies (Dimigen et al., 2012; Kornrumpf et al., 2016; Li et al., 2015; Niefind & Dimigen, 2016), we obtained clear activation differences at the temporal, parietal, and occipital regions, maximal over the left hemisphere, reflecting the early *preview positivity* effect (i.e., more positive voltage for identity preview conditions). These differences sustained through the 120-ms to 300-ms time window and into the 300-ms to 500-ms window also. Dimigen et al. (2012) identified an early preview effect in the first time window over occipito-temporal regions and a later effect between 300 ms and 500 ms over the midparietal electrodes. Clearly, the current findings replicate the early effect of Dimigen et al. (2012), and show that there are effects with a similar onset profile in left and right frontal areas as well. However, the present results show identity preview conditions to be more negative than X-string previews over centro-parietal regions of the scalp. This is consistent with the observation that in the X-string preview condition, pronounced orthographic processing is still ongoing between 300 ms and 500 ms compared with the identity preview condition (see Figure 5B). More research is needed to investigate the late preview effect during natural reading. Figure 4F represents the significant voltage differences between the random letter string and identity preview conditions. From Figure 4F, we again have positive activation differences over the right temporal, occipital, and parietal areas, presumably reflecting differences in how the target was visually processed post fixation onset when the preview was a letter string relative to the identity preview condition. At central locations there are negative voltage differences from fixation onset that persist through all of the time windows up to approximately 250-ms postfixation onset. As we suggested earlier, let us assume that these areas are associated with processing of stimuli beyond isolated letters. If this is the case, then these activation differences may well reflect processing associated with inhibition due to differences between the preview relative to the target in the letter string preview condition and reduced disruption due to feature overlap between target and preview in this condition. Voltage differences were also observed in the time window between 300 ms and 500 ms over parietal and occipital sites of the scalp. We believe this activation difference is associated with spill-over effects that derive from the target word.

In sum, our FRP data suggest that differences between the identity condition and the X-string preview condition are more sustained than counterpart differences between the identity and letter string preview conditions. The reason for this is likely that the X-string previews prevent readers from engaging in effective parafoveal processing. Because strings comprised of Xs are perceived as visually unusual, further processing of the type that would occur naturally for a parafoveal word is inhibited, and full identification of the target word can only be initiated subsequently when the target becomes available in the fovea. In this way, the X-string preview results in a delay to processing in the temporal windows we considered in relation to target word foveation. In contrast, letter string previews are processed, at least at an orthographic level, in the parafovea. Thus, when the target is fixated, preprocessing of the preview in relation to the target, leads to inhibition, due to conflicting information between parafoveal and foveal vision, but to a lesser extent, due to preactivation of visual features of the target.

### Foveal Lexical Frequency Effects

One of the benchmark findings in experimental psycholinguistics is that the frequency of occurrence of a word determines the time for its recognition (Rayner, 1998, 2009). Although its existence is undoubted and very well documented in eye movement experiments investigating reading, with respect to ERP and FRP experimentation, the nature and timing of this effect is unclear (Kretzschmar et al., 2015), and may be influenced by specific task circumstances (see Laszlo & Federmeier, 2014). Thus, as mentioned earlier, an aim of the present study was to further investigate this effect under natural reading conditions.

Our eye movement results showed a very clear word frequency effect. Low-frequency words led to significantly longer reading times than high-frequency words, and this effect increased from first fixation to gaze duration (10 ms difference between high-frequency and low-frequency words on first fixation duration, 14 ms on single fixation duration and 22 ms on gaze duration; see Table 2 and Table 3). The magnitude and time course of these effects is in line with the existing literature (e.g., Reingold, Reichle, Glaholt, & Sheridan, 2012) and demonstrate that lexical identification of a high-frequency word takes less time than lexical identification of a low-frequency word. This effect is unsurprising but reassuring. In addition, as can be seen in Table 3, we found a significant interaction between target lexical frequency and the parafoveal preview type for first and single fixation durations between the identity and random letter string conditions. The lexical frequency effect was more pronounced when participants received a valid preview in the parafovea (18 ms difference between high-frequency and low-frequency words on first fixation duration, 19 ms on single fixation duration) than when the parafoveal preview was a string of random letters (1 ms difference between high-frequency and low-frequency words on first fixation duration, 6 ms on single fixation duration). This result fits very neatly with our preview benefit findings reported earlier. Recall that a valid preview in the parafovea produced shorter target reading times than an invalid preview suggesting earlier initiation of target processing for valid previews. In line with this suggestion, the interactive effect of preview and target frequency were such that frequency effects were largest for first fixations on the target after a valid than an invalid preview, again, suggesting that processing of the target was initiated earlier when the preview was valid compared with invalid. Recently, Staub and Goddard (2018) suggested that word frequency might affect both early orthographic and late lexical stages of processing. According to their hypothesis, only the early stages (as reflected, e.g., by first fixation duration) might be dependent on whether early orthographic processing could be carried out parafoveally. If that is the case, this could explain why we found an interaction on the early (i.e., first and single fixation durations), but not later (i.e., gaze duration), eye movement measures. In the present experiment, readers could successfully retrieve the orthographic representation of the word from the parafovea in the valid preview condition (i.e., initiating orthographic processing of the correct orthographic representation of the word earlier), but once fixated, lexical access could be successfully carried out for both high-frequency and low-frequency words.

Interestingly however, FRPs time-locked to the fixation onset of the target and elicited by high-frequency words did not differ from the FRPs evoked by low-frequency words (see Figure 5 for associated waveforms and topographies). To be very clear, in our basic analyses of the FRPs at the target word, there was absolutely no statistically robust evidence of any effect of frequency in any time window or brain area that we analyzed. The lack of FRP frequency effects at the target word mirror exactly the lack of FRP frequency effects at the pretarget word. Note also that the lack of FRP frequency effects is in very direct contrast to the robust frequency effects that we obtained in the eye movement data at the target word. In line with the analyses we undertook for the pretarget word, to very directly determine whether there were any differences between processing of high- and low-frequency target word with and without a valid preview, we undertook an analysis in which we compared high- and low-frequency targets after a valid preview, and an analysis in which we compared high- and low-frequency targets after an invalid preview (both for letter-string previews and for X-string previews). Again, none of these statistical analyses revealed any significant effects of frequency (see Figure 5). Our results are consistent with Kretzschmar et al. (2015) who also failed to find any significant effect in the latency of the P200 and N400 components. Furthermore, in our analyses, we also examined the time window prior to 150 ms from fixation onset on the target word, and again, found no reliable FRP frequency effect. The current findings, as well as those of Kretzschmar et al. (2015) both derive from natural reading experiments, and are at odds with the results of studies using single word presentation methodology (e.g., Dambacher et al., 2006, 2012; Hauk et al., 2006; Sereno et al., 1998) and saccadic word list reading (Niefind & Dimigen, 2016). Kretzschmar et al. (2015) explained the lack of effect in agreement with the bidirectional coding account (Lotze, Tune, Schlesewsky, & Bornkessel-Schlesewsky, 2011; Tune et al., 2014). According to this approach, the N400 amplitude might reflect the extent to which bottom-up information and top-down predictions mismatch. Therefore, when words are presented within extensive context (either one word at the time or within a sentence), the effect of word frequency might not exert a very strong influence on the N400 component because under these circumstances, it is word predictability, rather than word frequency, that would act as a source of lexical expectation. Although this account might explain our null FRP effect of word frequency in the latest time window of analysis, it cannot fully explain the null results we observed over all the time windows analyzed. It is clear that there remains significant unanswered questions regarding the frequency effect in our FRP data. There is no question that a frequency effect occurred in this experiment. We can make this statement definitively on the basis of the eye movement data. We can also offer possible explanations in terms of the methodological differences between those studies that have successfully shown FRP frequency effects, and those that have not, though in our view, these explanations are not entirely satisfactory, and we can see no convincing reason why there should be no evidence of a frequency effect in our FRP data when such an effect is very apparent in the corresponding eye movement data. Accepting all of this, however, the fact remains that to date there have been only two studies to investigate frequency effects in FRPs under natural reading conditions (that of Kretzschmar et al., 2015, and the present study), and neither of these has provided any evidence for such an effect. Further research is required to develop our understanding of the experimental circumstances under which FRP frequency effects might be detectable during natural reading.

## Conclusions

In the current experiment, we simultaneously recorded participants’ eye movements and EEG signal during a natural sentence reading task. Because to date only a handful of studies have used the coregistration methodology to investigate reading under natural conditions, we considered effects previously unexplored (i.e., visual and orthographic PoF and preview effects) or only occasionally examined (i.e., lexical frequency effects) during natural reading of sentences.

Our results replicated the preview effects reported both in the eye movement (see Schotter et al., 2012 for a review) and FRP literature (i.e., RSVP-with-flanker-word presentation and saccadic reading studies; Dimigen et al., 2012; Kornrumpf et al., 2016; Li et al., 2015; Niefind & Dimigen, 2016). Previews that consist of words identical to the target lead to facilitation (or reduced cost) compared with X-string previews or random letter-string previews both during parafoveal and foveal processing of the target word. In addition, our findings demonstrate that previews comprised of X-strings activate different cognitive mechanisms compared to previews comprised of strings of letters. Previews comprised of X-strings appear as visually odd in the parafovea, inhibiting preprocessing of the preview string. This means the onset of processing is delayed until the target is directly fixated and disruption to reading occurs. In contrast, strings of random letters appear as previews that are more word-like in the parafovea. Thus, disruption is reduced and limited to processes beyond visual encoding of stimuli and processing of isolated letters (e.g., Carreiras et al., 2014). Inhibition occurs due to difficulties associated with the integration of inconsistent parafoveal and foveal information, but it is reduced because of the activation of features shared by the preview and target. We believe these findings contribute to the debate of costs and benefits associated with different types of preview (see Vasilev & Angele, 2017 for a review).

In addition, we consider that the raster diagrams (see Figure 4) presented in this article clearly show important aspects of the time course of the processes underlying natural reading. We acknowledge that only significant voltage differences are shown (at a significance level of .025 in each tail) and therefore, that other differences might occur but without reaching significance. Nevertheless, and for this very reason, we speculatively argue that the onsets of the differences we can observe in the raster diagrams represent the upper time limit of the onset of those effects, while the offsets the lower time limit of the duration of these effects. To be very clear, the period between the onset and offset of a significant effect in the raster diagrams represents an approximation of the minimum period during which differences in processing occurred. Certainly, we found the raster diagrams from this study to be very helpful in interpreting our findings, providing us with a comprehensive representation of activation differences at all scalp locations over extended time periods. With more studies and analyses of this type it should be possible to gain much greater insight into the nature and time course of neural correlates of natural reading processes.

Finally, the current study highlights the necessity to explore the timing of the word frequency effect under ecologically valid conditions. Neither our eye movement or FRP results provided evidence for extraction of word frequency from the parafovea—hence, providing no evidence to support parallel lexical processing. At the fovea, we replicated the well-established frequency effects in the eye movement data, but not in the FRPs. The reason why frequency effects did not appear in the FRP data for the present study is not clear, though again, we note that these effects also did not appear in the study reported by Kretzschmar et al. (2015). For now, our suggestion is that rapid automatized processes that occur in natural reading might lead to short-lived, reduced magnitude, effects compared with less natural reading situations. This said, it is clear that further coregistration experiments using natural reading are necessary to better understand the nature of FRP responses associated with lexical frequency effects in eye movements.

## Figures and Tables

**Table 1 tbl1:** Characteristics of Words Used in the Current Experiment

	Pretarget word 1	Target word 1				Post-target word 1
Measure	Length	HF	LF	Length	Position	Length
Mean	5.32	291.96	13.08	5.59	3.74	5.63
*SD*	.97	269.54	5.89	.49	.85	1.51
	Pretarget word 2	Target word 2				Post-target word 2
Mean	5.32	386.65	12.39	5.59	9.08	6.09
*SD*	.99	650.96	5.52	.49	1.18	1.65
*Note.* High frequency (HF) and low frequency (LF) are reported in terms of frequency per million. Length is measured in number of characters. Position refers to the number of words that preceded the target word in the sentence.						

**Table 2 tbl2:** Means and Standard Deviations of the Fixation Time Measures (in Milliseconds) for Pretarget and Target Words

Measure	HF Identity	HF String	HF X	LF Identity	LF String	LF X
	Pretarget word					
FFD	228 (62)	228 (66)	238 (71)	232 (68)	229 (61)	236 (70)
SFD	231 (56)	229 (62)	240 (67)	237 (72)	234 (61)	243 (70)
GD	259 (93)	263 (101)	280 (105)	259 (94)	263 (96)	280 (106)
	Target word					
FFD	235 (69)	284 (84)	280 (80)	253 (71)	285 (88)	289 (84)
SFD	244 (70)	308 (75)	308 (68)	263 (67)	314 (78)	325 (68)
GD	268 (100)	327 (101)	327 (84)	289 (105)	346 (114)	353 (103)
*Note*. Means and standard deviations have been calculated by subject. FFD = first fixation duration; SFD = single fixation duration; GD = gaze duration; HF = high-frequency.						

**Table 3 tbl3:** Fixed Effect Estimates From the Linear Mixed-Effects Models on the Eye Movement Data for Pretarget and Target Words

Factor	First fixation duration				Single fixation duration				Gaze duration			
	*b*	*SE*	*t*	Sign.	*b*	*SE*	*t*	Sign.	*b*	*SE*	*t*	Sign.
	Pretarget word											
Intercept	5.398	.021	262.138	***	5.415	.022	246.935	***	5.511	.025	220.654	***
Frequency	.002	.008	.200		.017	.012	1.442		−.003	.010	−.338	
String-Identity	.002	.010	.145		.012	.014	.885		.014	.012	1.124	
X-String	.023	.013	1.820	+	.023	.016	1.478		.054	.016	3.391	**
Identity-X	−.024	.014	−1.774	+	−.035	.017	−2.037	*	−.068	.014	−4.797	***
Frequency × String-Identity	<.001	.019	.008		.015	.025	.587		.010	.024	.415	
Frequency × X-String	−.014	.023	−.606		−.013	.029	−.453		−.002	.024	−.088	
Frequency × Identity-X	.014	.022	.644		−.002	.029	−.056		−.008	.026	−.311	
	Target word											
Intercept	5.548	.018	312.756	***	5.630	.020	275.509	***	5.696	.020	281.832	***
Frequency	.032	.009	3.604	***	.050	.013	3.967	***	.063	.010	6.644	***
String-Identity	.152	.016	9.657	***	.213	.023	9.130	***	.204	.017	12.004	***
X-String	<.001	.017	.016		.011	.023	.502		.013	.022	.592	
Identity-X	−.153	.019	−8.083	***	−.225	.024	−9.247	***	−.217	.022	−10.013	***
Frequency × String-Identity	−.069	.021	−3.314	**	−.056	.026	−2.103	*	−.027	.024	−1.147	
Frequency × X-String	.022	.023	.955		.026	.029	.876		.011	.027	.412	
Frequency × Identity-X	.047	.024	1.991	+	.030	.030	1.001		.016	.031	.519	
+ *p* < .1. * *p* < .05. ** *p* < .01. *** *p* < .001.												

**Table 4 tbl4:** Summary of the Statistical Differences Between Preview Conditions Observed in the FRP Data With Cluster-Based Permutation Tests

Comparison	Time window							
	0 ms–70 ms		70 ms–120 ms		120 ms–300 ms		300 ms–500 ms	
	Cluster	*p*-value	Cluster	*p*-value	Cluster	*p*-value	Cluster	*p*-value
	Pretarget word							
X-Identity	NA	NA	1 positive	<.004	1 positive	<.004	1 positive	<.001
	NA	NA	0 negative		1 negative	<.020	1 negative	<.003
X-String	NA	NA	1 positive	<.001	1 positive	<.001	1 positive	<.001
	NA	NA	1 negative	<.002	1 negative	<.001	1 negative	<.001
String-Identity	NA	NA	1 negative	<.020	1 negative	<.008	1 negative	<.002
	Target word							
X-Identity	1 positive	<.004	1 positive	<.001	1 positive	<.001	1 positive	<.001
	0 negative		0 negative		1 negative	<.001	1 negative	<.001
X-String	1 positive	<.001	1 positive	<.001	1 positive	<.001	1 positive	<.001
	1 negative	<.001	1 negative	<.005	1 negative	<.001	1 negative	<.001
String-Identity	1 positive	<.020	1 positive	<.005	1 positive	<.009	1 positive	<.020
	1 negative	<.001	1 negative	<.001	1 negative	<.001	0 negative	
*Note*. FRP = fixation-related potentials.						

## Footnotes

1 Given the formative state of the existing literature on coregistration, we wanted to adhere to standard experimental techniques that have been used in the past. For this reason, we used stimuli of the form that have been widely used in existing eye movement preview studies, and manipulated word frequency only, without manipulating other factors (e.g., orthographic neighborhood, morphological complexity, bigram or trigram frequencies).

2 Spatial adjacency of electrodes was defined with the method “triangulation” of the “ft_prepare_neighbours” function from the Matlab toolbox *FieldTrip* (Oostenveld et al., 2011).

3 In a diametrically opposite location to the P1 component, that is, at a frontal, slightly left lateralized location, there is an area of negative activation. We consider that this activation reflects the polarity of the brain activity that arises as a consequence of the strong posterior P1 positivity.

4 We note that word frequency effects at the pre-target word were absent even though there was a significant difference in the bi-gram frequency of the high (bigram lexical frequency by position for HF words: *M* = 3227.08, *SD* = 1401.19) and the low (bigram lexical frequency by position for LF words: *M* = 2920.01, *SD* = 1386.04) frequency target words. Had we obtained robust frequency effects at the pretarget word, then the question of whether those effects were driven by the frequency of the full word, or instead by the frequency of the initial bigrams would be very important.
